# The neuroprotective effects of hesperidin and diosmin in neurological disorders via targeting various signaling pathways

**DOI:** 10.22038/ijbms.2025.81908.17719

**Published:** 2025

**Authors:** Nikoo Abharian, Noushin Nikray, Afsoon Feizi, Marjan Talebi, Hamed Shafaroodi, Mehrdad Faizi

**Affiliations:** 1 Department of Pharmacology and Toxicology, School of Pharmacy, Shahid Beheshti University of Medical Sciences, Tehran, Iran; 2 Faculty of Pharmacy and Pharmaceutical Sciences, Tehran Medical Sciences, Islamic Azad University, Tehran, Iran; 3 Department of Pharmacognosy, School of Pharmacy, Shahid Beheshti University of Medical Sciences, Tehran, Iran; 4 Department of Pharmacology, School of Medicine, Tehran University of Medical Sciences, Tehran, Iran

**Keywords:** Alzheimer’s disease, Diosmin, Flavonoids, Hesperidin, Neuropathy, Neuroprotective agents, Signal transduction

## Abstract

Diosmin and hesperidin, two flavonoid glycosides derived from citrus plants, offer numerous health benefits, whether used alone or in combination. They can mitigate neurodegenerative symptoms and reduce vascular and microbial inflammations, improving cardiovascular health and modulating immunity and anti-oxidant properties. Neuroinflammation and oxidative stress are associated with numerous neurological disorders. This study aims to review the neuroprotective potentials of diosmin and hesperidin, focusing on their effects on oxidative stress and inflammatory conditions. A scientific literature search was conducted using specific keywords in various databases, including PubMed, Web of Science, Google Scholar, Embase, and Scopus.Diosmin and hesperidin demonstrated protective effects in managing a variety of neurological disorders, such as epilepsy, neuropathy, depression, anxiety, insomnia, autism, glioblastoma, traumatic brain injury, cerebral ischemia-reperfusion, Huntington’s disease, Alzheimer’s disease, Parkinson’s disease, neurotoxicity, and multiple sclerosis through the regulation of various signaling pathways. Hesperidin and diosmin exhibit promising neuroprotective effects that underscore their potential as natural multi-targeted therapeutic agents for treating neurological disorders through diverse cascades.

## Introduction

Due to the epidemiological burden, various neurological disorders such as epilepsy, depression, anxiety, insomnia, neuropathy, autism, glioblastoma, cerebral ischemia-reperfusion, traumatic brain injury, Huntington’s disease, Alzheimer’s disease, Parkinson’s disease, and multiple sclerosis have been widely investigated. Numerous studies have emphasized the significance of critical factors such as oxidative stress and neuroinflammation in the pathogenesis of these diseases (1). Natural products such as flavonoids possess biological properties that selectively target the biochemical mechanisms and signaling pathways underlying the pathogenesis of nervous system-associated diseases (2). Flavonoids are natural substances found in plants’ fruits, leaves, and flowers. They are categorized into various groups depending on their structure and the amount of hydroxylation and oxidation. Examples of flavonoids include flavonols, flavanones like hesperidin, flavanols, flavones like diosmin, and anthocyanins. Flavonoids can exist in a glycosylated form or as aglycones (3). Flavonoids are recognized for their range of characteristics, including anti-oxidative, anti-inflammatory, and anticancer properties (4).

Diosmin is produced through the oxidation of hesperidin. Diosmin and hesperidin differ in that diosmin has a double bond between two carbon atoms in the C ring, whereas hesperidin does not have this feature (5). Findings of a double-blind phase III clinical trial have shown that using micronized diosmin in doses up to 2000 mg daily for four months is safe (6). In *in vivo* studies on Sprague-Dawley rats, the average lethal dose (LD_50_) of hesperidin is 4837.5 mg/kg, and the low observed adverse effect level is 1000 mg/kg; this indicates the superb safety profile of hesperidin (7). In toxicity studies, hesperidin shows a high safety profile, which is estimated to be more than 2 g/kg orally (8). In animal studies, daily intragastrically of 100 mg Daflon^®^, consisting of micronized flavonoids, diosmin (450 mg), and hesperidin (50 mg), in rats was not associated with side effects (9). The results of toxicity studies on humans also indicate the safety of Daflon^®^-500 mg orally (10). Various pathological factors, such as oxidative stress and inflammation, are responsible for the progression of neurological disorders. Because of the multiple functions of natural compounds, they can be beneficial in managing these physiological conditions. Therefore, the present review aimed to elaborate on the promising effects of diosmin and hesperidin, alone or in combination in various neurological disorders by considering the involved signaling pathways.

## Methodology

This review extracts and discusses findings from various studies examining the neuroprotective effects of diosmin and hesperidin, both applicated alone and in combination. The review does not impose restrictions on publication dates. A comprehensive search was conducted using scientific databases, including PubMed, Web of Science, Google Scholar, Embase, and Scopus. Specific keywords utilized in this search included “diosmin”, “hesperidin”, “Daflon”, “neuro*”, “neurological disorder”, “epilepsy”, “neuropathy”, “depression”, “anxiety”, “insomnia”, “autism”, “glioblastoma”, “traumatic brain injury”, “cerebral ischemia-reperfusion”, “Huntington’s disease”, “Alzheimer’s disease”, “Parkinson’s disease”, “neurotoxicity”, and “multiple sclerosis”. The search was performed until May 28, 2024, and relevant studies were included in this review. 

## Anti-oxidative effects of hesperidin and diosmin

Under usual physiological conditions, each cell creates a specific amount of ROS through various processes. When ROS levels are within normal limits, the cell’s anti-oxidant defense system is activated, preventing significant damage. However, excessive ROS within the cell can damage protein functions, membrane fluidity, and nucleic acids. Radical species damage all cell components, including lipids, proteins, and DNA. Because of high oxygen demand, susceptibility of lipid cells to peroxidation, and weak anti-oxidant defense, the brain organ is vulnerable to ROS. According to studies, neurodegenerative disorders such as Alzheimer’s and Parkinson’s diseases are closely related to oxidative stress and may be managed by using anti-oxidant agents (11).

Nrf2 is a transcription factor that can activate the transcription of enzyme genes involved in anti-oxidant defense. In the conditions of oxidative stress, the transcription factor Nrf2 enters the nucleus and, by binding to the promoter region of its target genes, leads to the transcription of genes (for example, superoxide dismutase (SOD) and glutathione peroxidase enzymes). The enzymes reductase, catalase (CAT), and heme oxygenase 1 (HO-1) substantially protect cells against oxidative stress (12).

NF-κB is a transcription factor involved in the inflammatory response, immunity, cell proliferation and differentiation, autophagy, and apoptosis. Increased ROS and oxidative stress considerably activate the NF-kB pathway (13). Hesperidin has the potential to demonstrate neuroprotective properties by inhibiting the NF-κB cascade and activating the Akt/Nrf2 signaling pathway to reduce inflammation and oxidative stress (14). Hesperidin has displayed promising anti-oxidant activities through scavenging ROS, inhibiting malondialdehyde (MDA) production, increasing SOD and glutathione (GSH) expression, and promoting the endogenous anti-oxidant defense system, especially Nrf2 and HO-1 (15). Hesperidin may function as a neuroprotective agent by activating peroxisome proliferator-activated receptor gamma (PPAR-γ), and PPAR-γ up-regulation reduces oxidative stress and inflammation (16).

Numerous studies have shown the anti-oxidant properties of diosmin (5, 17, 18). Diosmin can be beneficial in combating oxidative stress caused by H_2_O_2_ and can reduce the level of MDA. Diosmin can also increase anti-oxidant enzymes and GSH levels and reduce lipid peroxidation (18). Therefore, diosmin may have a protective role against disorders related to oxidative stress.

## Anti-inflammatory effects of hesperidin and diosmin

Inflammation is a kind of innate immune system response in conditions of cell damage by various factors such as microorganisms, trauma, necrosis, chemical or physical agents, and metabolic stress. Inflammatory responses occur to restore homeostasis and appropriate tissue function (19). Acute inflammation is the body’s primary response to an inflammatory agent or infection. In infection and tissue injury, the flow of plasma and leukocytes, including neutrophils and macrophages, increases. Additionally, leukocytes release various chemical substances such as histamine, cytokines, chemokines, lipids, and more. When the inflammatory response is not terminated, inflammation persists and progresses into a chronic state. Chronic inflammation is associated with metabolic syndrome, atherosclerosis, cancer, and diverse neurodegenerative disorders (20).

Hesperidin exhibits pronounced anti-inflammatory activity by inhibiting the release of pro-inflammatory cytokines, such as IFN-γ and IL-2, and blocking inflammatory responses activated by IL-1β and the NF-κB signaling cascade (21). Due to its anti-oxidant and anti-inflammatory effects, hesperidin has been effective in improving symptoms in animal models of neurological diseases such as Alzheimer’s disease, Parkinson’s disease, Huntington’s disease, multiple sclerosis, brain ischemia-reperfusion injury, and traumatic injury in the central nervous system (22). Hesperidin can significantly inhibit the production of nitric oxide (NO) and pro-inflammatory mediators, including TNF-α and IL-1β, in the RAW246.7 macrophage cell line (23). Therefore, studies indicate that hesperidin suppresses inflammation in the brain and other body parts by reducing pro-inflammatory cytokines, helping to scavenge radicals, and restoring cell function (4). Inflammatory processes with increased expression of COX-2 and iNOS play a significant role in the neurodegeneration cascade. The transcription factor, NF-κB, causes the transcription of pro-inflammatory cytokines that play an essential role in neuroinflammation and neurodegeneration. NF-kB causes the transcription of pro-inflammatory genes such as COX-2, iNOS, and TNF-α (24). In the intracerebroventricular-streptozotocin mouse model for memory impairment, hesperidin, with its anti-inflammatory properties, prevents nuclear translocation of NF-kB and the generation of COX-2, iNOS, and astrocyte activation (25).

Diosmin can inhibit many inflammatory mediators, such as TNF-α, COX-2 (26), and myeloperoxidase (MPO). The MPO enzyme is abundant in neutrophils and indicates neutrophil infiltration. Levels of MPO can indicate inflammation and oxidative stress (17). Pro-inflammatory arachidonic acid derivatives activate neutrophils and subsequently produce ROS in inflammatory tissue. Hesperidin and diosmin can inhibit the synthesis and activity of these derivatives (27). Through its anti-inflammatory activity, diosmin inhibits the activation of NF-κB, leading to the inhibition of inflammatory genes and the reduction of TNF-α and iNOS expression (28). 

## Protective effects of hesperidin and diosmin in neurological disorders

**Figure 1:**
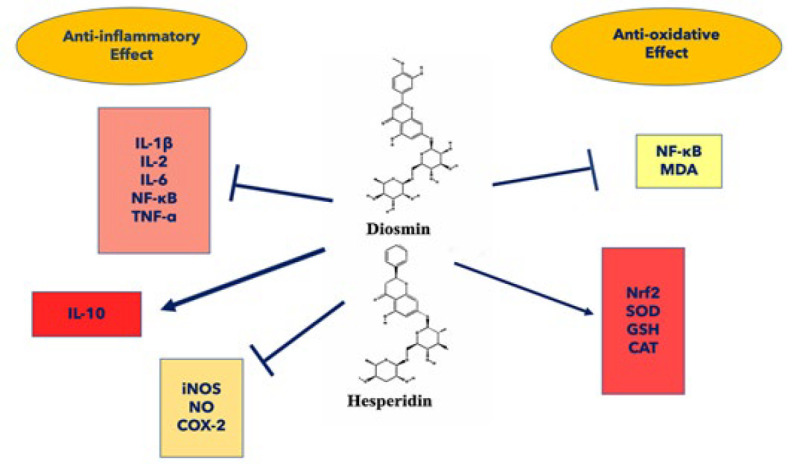
Anti-inflammatory and anti-oxidant effects of diosmin and hesperidin in neurological disorders

The neuroprotective targets and attributed intracellular mechanisms of diosmin and hesperidin are shown in Figure 1.

The figure illustrates the protective mechanisms through which the flavonoids diosmin and hesperidin exert their anti-oxidant and anti-inflammatory effects, both *in vivo* and in vitro. These compounds enhance anti-oxidant defenses by increasing the levels of anti-oxidant enzymes such as CAT and SOD, while simultaneously reducing levels of ROS. Additionally, diosmin and hesperidin exhibit anti-inflammatory properties by decreasing the production of inflammatory cytokines, including IL-6, IL-1β, TNF-α, and NO, while promoting the level of the anti-inflammatory factor IL-10. Arrows with blunt tips indicate inhibition, whereas directional arrows represent activation.

## Effects of hesperidin and diosmin on Alzheimer’s disease

Alzheimer’s disease (AD) is a progressive neurodegenerative disease associated with impaired memory, language function, spatial perception, and personality disorders. The exact cause of the disease is not known (29). The most proposed hypotheses in the pathophysiology of AD include cholinergic imbalance, oxidative stress, defects in the cellular anti-oxidant defense system, disruption of the mitochondrial membrane potential, excitotoxicity, and inflammation. Studies show a connection between inflammation, oxidative stress, and AD. Disruption of cortical cholinergic transmission leads to Amyloid-β (Aβ) pathology and an increase in phosphorylation of tau protein (30). In the blood and cerebrospinal fluid (CSF) of patients with Alzheimer’s disease, increased levels of pro-inflammatory cytokines are seen, and this indicates the importance of inflammation in the development of Alzheimer’s disease. Cytokines such as TNF-α, IL-10, IL-1β, and type I interferon are strongly associated with AD (31). Therefore, anti-cholinesterase, anti-inflammatory, and anti-oxidant treatments can benefit disease management (30).

Hesperidin can improve neurogenesis in the hippocampus of *5xFAD* mice and reduce Aβ accumulation and memory dysfunction (29). In the animal model of AD, valproic acid-induced memory-impaired rats, hesperidin (100 mg/kg by oral gavage for 21 days) can be effective in improving memory and learning ability by facilitating hippocampal synaptogenesis through up-regulating nerve growth factor levels (32). In healthy adult mice, hesperidin (100 mg/kg intraperitoneally) can improve recognition memory through increasing hippocampal synapse formation and TGF-β1 signaling. In brain development and function (including astrocyte production and synapse formation), TGF-β1 is involved (33). In APPswe/PS1dE9 transgenic mice, hesperidin (100 mg/kg) effectively lessened mitochondrial dysfunction caused by Aβ and cognitive disorders. Therefore, hesperidin may be promising in managing AD (34).

The results of animal studies show that diosmin has neuroprotective effects and can improve cognitive functions. The findings show that diosmin (50 and 100 mg/kg intraperitoneally) effectively ameliorates brain functions such as memory, motor coordination, and gait in quinolinic acid-intracerebroventricular-treated rats through improving mitochondrial function and anti-oxidant effect (35). Diosmin (50, 100 mg/kg for seven days) can protect the cholinergic system from scopolamine-induced memory impairment by inhibiting NF-κB signaling pathways, reducing TNF-α levels in the hippocampus, and improving memory and motor function (36). Through inhibiting glycogen synthase kinase-3, diosmin can reduce brain Aβ oligomer levels in the 3xTg-AD mouse model (37) and a transgenic mouse model (Tg2576) (38). It also reduces tau hyperphosphorylation and cognitive disorders in a mouse model of 3xTg-AD (37). In APP/PS1 transgenic mice, diosmin has caused the destruction of Aβ and improved cognitive impairment and memory deficit by activating the aryl hydrocarbon receptor (AhR) and increasing the expression of Neprilysin (NEP). NEP is an essential enzyme in the degradation of Aβ in the brain and the periphery. AhR is abundantly expressed in the central nervous system (CNS) and has a detoxification function. In AhR knockout mice, movement defects and changes in myelin structure are discerned (39). Therefore, according to the results obtained from studies, diosmin can usefully restore memory and control dementia (36).

## Effects of hesperidin and diosmin on Parkinson’s disease

Progressive Parkinson’s disease (PD) is a common age-related neurodegenerative disorder. The loss of dopaminergic cells of the nigrostriatal pathway is associated with motor symptoms, including resting tremor, postural instability, bradykinesia, rigidity, and gait disturbance. The loss of dopaminergic neurons causes dysfunction of the basal ganglia, which participate in the initiation and execution of movements. Also, this disease is associated with non-motor symptoms, such as depression and anxiety (40). Although the exact etiology of PD is still unknown, aging, environmental and genetic factors, oxidative stress, and neuroinflammation are feasibly involved in PD pathogenesis (41).

Hesperidin is a neuroprotective agent against PD, AD, stroke, and Huntington’s disease. It also has antidepressant, anticonvulsant, and anxiolytic effects (42). Hesperidin (through anti-oxidant and DA-increasing mechanisms) can exert various protective roles in PD models. Hesperidin (50 mg/kg) for 28 days, in an animal model of Parkinson’s disease induced by 6-hydroxydopamine (6-OHDA), can effectively prevent the depletion of DA and its metabolites, 3,4-dihydroxyphenylacetic acid and homovanillic acid (43). Hesperidin (50 mg/kg orally for 28 days), in the 6-OHDA Model of PD, can also reduce the loss of dopaminergic innervation in mice striatum, which is related to the anti-inflammatory and neurotrophic enhancing mechanism of hesperidin (40). Hesperidin has been beneficial in zebrafish larvae against oxidative stress-induced Parkinsonism and 6-hydroxydopamine (6-OHDA) toxicity. 6-OHDA can cause oxidative stress and destroy dopaminergic neurons. In SH-SY5Y cells, hesperidin enhances the anti-oxidant defense mechanisms, increases the mitochondrial membrane potential, and significantly reduces intracellular ROS levels. As a result, hesperidin helps to improve cell damage caused by 6-OHDA (42). 3,4-Dihydroxyphenylacetic acid and striatal dopamine levels were restored in 6-hydroxydopamine-damaged rats by hesperidin (44). Hesperidin (50 mg/kg for 24 days) acts more effectively than L-dopa in the 6-OHDA PD rat model, and simultaneous administration of hesperidin with L-dopa improved this drug’s bioavailability (45). In the SH-SY5Y cellular model of PD, hesperidin can advantageously prevent and treat PD through its effect on intracellular calcium homeostasis and reducing the concentration of intracellular calcium and its receptors (46). Hesperidin may effectively function in striatal dopaminergic denervation, neuroinflammatory reactions, and neurotrophic deficits. Therefore, the neuroprotective properties of hesperidin can have potential therapeutic potential in treating PD (44).

Agricultural toxins can cause parkinsonism, and one example is the neurotoxin rotenone. Rotenone can mimic features of PD, including dopaminergic neuronal death, inflammation, mitochondrial dysfunction, and oxidative stress. Diosmin had a neuroprotective effect on rotenone-induced apoptosis in SH-SY5Y cells. The anti-apoptotic effects of diosmin can be related to the anti-oxidant properties and stability of mitochondrial membrane potential (47). Diosmin (200 mg/kg orally for four weeks) prevented movement disorder and tissue damage and decreased tyrosine hydroxylase expression in the animal model of Parkinson’s using rotenone. It also reduces NF-κB expression and TNF-α levels (28). Therefore, diosmin can be valuable for the treatment of neurodegenerative disorders such as PD (47).

## Effects of hesperidin on Huntington’s disease

Progressive Huntington’s disease (HD) is a neurological disorder associated with cognitive, emotional, and motor abnormalities (48). In this disease, the repetitive sequence of cytosine, adenine, and guanine is observed in the Huntingtin gene (HTT), which can cause mutations. Oxidative stress may be activated by independent primary causes and is involved in the pathology of neurodegenerative diseases such as Huntington’s. Oxidative stress can play a role in aggravating the disease, leading to protein misfolding and the formation of the clumps that stop nerve transmission. Also, NF-κB directly binds to the HTT gene promoter and increases its transcription, which increases mutant HTT expression in striatal neurons (49).

The neurotoxin 3-nitropionic acid (3-NP) produces HD-like symptoms in animals and humans *via* irreversibly inhibiting the enzyme succinate dehydrogenase (complex-II) in the electron transport chain, and it can induce the Huntington’s disease model in animal studies (50). Hesperidin (100 mg/kg orally for five days) has anti-oxidant and anti-inflammatory activity that can have a neuroprotective effect against HD-like manifestations caused by 3-NP by maintaining the mitochondrial and nuclear membranes and the cerebral cortex normal vessels and reducing the expression of iNOS in the cortex, hippocampus, and striatum (48). Hesperidin (100 mg/kg orally for 14 days) increases oxidative defense in the striatum and affects cell energy reserves by regulating the NO pathway (50). The results show that hesperidin (100 mg/kg) in the rat model of HD caused by quinolinic acid for 21 days inhibits the activation of microglial cells. Since the microglial pathway is related to Huntington’s, hesperidin can have protective effects against this disease (51).

## Effects of hesperidin and diosmin on brain tumors

According to US cancer statistics for 2023, 23890 cases of brain tumors and other nervous system tumors and 18.020 deaths were estimated in 2023 (52). Glioblastoma multiforme (GBM) progresses rapidly and has a poor prognosis. Despite evident advances in glioblastoma treatments, the 5-year survival rate is ˂10%. Considering the challenges in the treatment of GBM, there is an urgent need for drugs and therapeutic approaches in the treatment of glioblastoma (53). In an in vitro study on the U-87 glioblastoma cell line, hesperidin (10 µM and 25 µM) can have anti-apoptotic and anti-proliferative effects by reducing caspase3 activity (54).

Diosmin can stop the cell cycle in the G_1_ phase and inhibit the growth of GBM cells. In addition, diosmin prevents the migration and invasion of GBM cells by suppressing the epithelial-mesenchymal transition (EMT-like) process. In EMT, epithelial cells acquire a mesenchymal phenotype, which increases cell motility and resistance to genotoxic agents. Diosmin induces cell cycle inhibition at the G_1_ phase in human glioblastoma cell lines GBM8401 and human LN229, which is not associated with drastic toxicity compared to the control astrocyte line SVG-p12 (55). The results of investigating the antitumor function of diosmin on glioblastoma cell lines (GBM95, GBM02, and U87MG glioblastoma cells) show that diosmin can be promising in the treatment of glioblastoma (56).

## Effects of hesperidin and diosmin on traumatic brain injury

Millions of people around the world suffer from neurobehavioral and cognitive abnormalities caused by traumatic brain injury (TBI) (22). TBI is associated with a series of events, including excitatory neurotransmitter release, inflammatory cytokine activation, and free radical formation, and each event contributes to the death of nerve cells (57).

In TBI, the expression of inflammatory factors (IL-1β and TNF-α) and oxidative stress increase, and the brain-derived neurotrophic factor (BDNF) level decreases in the mice hippocampus. Hesperidin (50 mg/kg for 14 days) reduces MDA as an indicator of oxidative stress and pro-inflammatory cytokines (IL-1β and TNF-α) and increases hippocampal BDNF levels in the TBI mouse model (22).

Diosmin (100 mg/kg for seven days before TBI induction) can prevent the increase of hippocampal TNF-α, improve neurobehavior, and repair BBB. Therefore, diosmin is anti-inflammatory in the TBI model (induced by Marmarou’s method) (58). 

## Effects of hesperidin and diosmin on cerebral ischemia-reperfusion (I/R) injury

Stroke is a common cause of disability and death in humans. Ischemic cerebral stroke involves the initial interruption of blood flow and reperfusion, which causes inadequate delivery of oxygen and glucose to the cells (59). Stroke causes defects in different areas of the brain, which causes a wide range of emotional, behavioral, and cognitive disorders. Oxidative stress, neuroinflammation, excitotoxicity, and mitochondrial dysfunction play a role in stroke (60).

Hesperidin (100 mg/kg for ten days) can amend stroke-induced neurodegeneration. In the stroke mouse model (C57BL/J6 mice), hesperidin had a beneficial effect on the anti-oxidant defense system and changes in lipid peroxidation levels, nitrite concentration, GSH, SOD, and CAT activities (61). Hesperidin (50 and 100 mg/kg for seven days) in the cerebral I/R injury model by the Jingtao method in rats has protective effects against cerebral ischemic reperfusion injury and memory dysfunction. This protective effect of hesperidin may be related to the nitric oxide signaling pathway (60).

Diosmin (50 mg/kg or 100 mg/kg orally for six days) reduced neurodeficiency and cerebral edema in a mice tMCAO model of stroke-induced brain damage. Diosmin can augment the number of Nissl-positive cells and the expression of Bcl-2 while diminishing the expression of Bax, thereby exhibiting an anti-apoptotic impact in the context of cerebral I/R (62). Activation of pSTAT3 in neurons and astrocytes following ischemic brain damage can be beneficial (63). Diosmin can activate the JAK2/STAT3 signaling pathway, thereby improving cerebral ischemia-reperfusion injury (62).

## Effects of hesperidin and diosmin on neuropathic pain

Neuropathic pain exerts its influence on numerous individuals across the world. It leads to experiencing a chronic, painful, and debilitating condition that disrupts daily activities (64). After nerve damage, destructive changes occur in the damaged neurons and along the pain-modulating and descending pathways in the CNS. This leads to tactile allodynia and hyperalgesia, which can be attributed to structural alterations resulting in increased transmission from Aβ fibers. Consequently, non-painful stimuli are conveyed to specific nociceptive neurons in the dorsal horn (65). Following peripheral nerve damage, spinal cord glial cells, including astrocytes, microglia, and oligodendrocytes, are activated. The spinal cord plays a role in regulating the peripheral input to the CNS. Pro-inflammatory mediators are released with the activation of glial cells, and pain is increased by nerve transmission. Cytokines released from glial cells stimulate nociceptors. Finally, the activity of spinal cord nociceptive neurons increases (central sensitization), and the activation threshold of nociceptive neurons decreases (peripheral sensitization) and can cause the spontaneous firing of nociceptive receptors (66). The neuroinflammatory response can cause peripheral and central neuropathic pain. TNF-α, IL-1β, and IL-6 play a considerable role in the pathogenesis of neuropathy (67).

In the context of neuropathy, up-regulation of COX-2, iNOS, TNF-α, and IL-1β *via* the NF-κB and MAPK signaling cascades induces the infiltration of inflammatory cells, including neutrophils and monocytes, into the sciatic nerve and spinal cord of neuropathic mice. Hesperidin (100 mg/kg) can reduce the expression of these factors in chronic constriction injury (CCI) rats (67). Hesperidin (50 mg/kg for 14 days) prevents pain signaling and abnormal hyperalgesia in CCI rats by inhibiting P2X3 expression in DRG neurons. P2X3 receptors are involved in the initiation and progression of chronic neuropathic pain (68).

Diosmin (1 or 10 mg/kg intraperitoneally for seven days in CCI mice) can target the production of hyperalgesia molecules and glial cell activation. Diosmin has an analgesic effect by activating the signaling pathway of NO/cGMP/PKG/ATP-sensitive potassium channels, inhibiting the expression of hyperalgesia cytokines, and activating glial cells. Diosmin inhibits CCI-induced mRNA expression of IL-1β, TNF-α, IL-33, ST2, Iba-1, and Olig2 in the spinal cord, which is related to its analgesic effect. Diosmin inhibits the production of TNF-α and plays a role in neuroprotection with its anti-apoptotic effect (66).

Hesperidin (100 mg/kg intraperitoneally) can improve thermal and mechanical hyperalgesia in the CCI model in rats, and this effect is intensified when used simultaneously with diosmin (10 mg/kg intraperitoneally). These effects can be related to the participation of dopaminergic, GABAergic, and opioidergic neurotransmission (69).

## Effects of hesperidin on epilepsy

Epilepsy is a persistent neurological condition characterized by complex etiologies. Abnormal electric discharge usually occurs because of different perinatal and postnatal injuries of neuronal circuits (70). Neuroinflammation is seen in various CNS disorders, such as autoimmune disorders and epilepsy, and can play a role in causing seizures and their continuation. Following a seizure, the level of pro-inflammatory factors such as IL-1β, IL-6, and TNF-α increases in the brain (71). The generation of free radicals and the lessening of anti-oxidant defense are observed in the brain after seizures. Increased production of free radicals leads to DNA and protein damage, tissue damage, inflammation, and apoptosis. Evidence shows that neuroinflammation and seizures are directly related. Also, oxidative stress and nerve damage in patients with epilepsy intensify seizures (70).

During seizures, the levels of pro-inflammatory cytokines in the brain increase, and inflammatory signaling pathways, such as toll-like receptor 4 (TLR4) and NF-κB, are activated. The results have shown that hesperidin (100 mg/kg) has protective effects on febrile seizures (hyperthermia-induced febrile seizures) through its anti-inflammatory and anti-oxidant properties and the down-regulation of TLR4 (71). The results of studies show that hesperidin (200 mg/kg 30 min before PTZ administration) can delay the onset of seizures. The combination of hesperidin (100 mg/kg) with diazepam or gabapentin had a more protective effect compared to diazepam and gabapentin alone (72).

## Effects of hesperidin on multiple sclerosis

Multiple sclerosis (MS) is a chronic progressive autoimmune inflammatory disease of the CNS associated with demyelination, axonal degeneration, and loss of motor function. The precise underlying mechanisms of this disease are inconspicuous, but a complex interaction between genes and environmental factors is probably involved. In this disease, oligodendrocytes and myelin sheath are ruined in the CNS. Activating macrophage/microglial cells, differentiating neural antigen-specific Th1 cells, and releasing inflammatory cytokines in the CNS are complex processes that lead to CNS demyelination (73).

Overexpression of pro-inflammatory cytokines such as IFN-γ, TNF-α, IL-6, and IL-17, and reduction of anti-inflammatory cytokines such as IL-4, IL-10, and TGF-β are associated with CNS inflammation and demyelination. Hesperidin (50–200 mg/kg for 25 days orally) in the myelin oligodendrocyte glycoprotein (MOG)-induced mouse model of MS can reduce the expression of pro-inflammatory cytokines (IL-6, IL-17, and TNF-α) and increase the expression of anti-inflammatory cytokines (IL-10 and TGF-β). Also, hesperidin can prevent the proliferation of auto-reactive T cells and penetration into the CNS. Hesperidin may reduce demyelination in the CNS in a dose-dependent manner in the C57BL/6 mice (73). The experimental MS mouse model of autoimmune encephalomyelitis (EAE) model in C57BL/J6 mice is associated with lipid peroxidation and suppression of enzymatic and non-enzymatic anti-oxidants (74). Hesperidin (50 mg/kg for seven days subcutaneously) has a beneficial effect in reducing these manifestations and attenuating the oxidative damage and tissue changes of the cerebral cortex induced by experimental allergic encephalomyelitis (51, 74).

## Effects of diosmin on autism spectrum disorder

Autism spectrum disorder (ASD), a neurodevelopmental disorder, is a complex condition that is associated with deficits in social interaction, language and communication abilities, and repetitive behaviors (75). Maternal immune activation through chronic induction of proinflammatory pathways that begin in the utero and increased maternal circulating cytokines in response to infection during pregnancy are hypotheses for the development of autism that have been proposed in recent years. Neuropathological and behavioral deficits in autism can be related to these immune and cytokine responses in the critical developmental period (76). ASD may be associated with maternal or infant atopic diseases, such as asthma, eczema, and food allergies. In this situation, mast cells produce inflammatory and vasoactive mediators that increase BBB permeability. An enhancement in the level of NF-κB in the brain, CSF, and serum of some patients and an increase in the expression of the inflammatory factors IL-1β, IL-6, 1L-17, and TNF-α have been observed in some other patients (77).

JAK2/STAT3 expression induced *via* IL-6 augments in the brain of children with autism. Treatment with diosmin (10 mg/kg orally) in IL-6/MIA adult offspring through preventing the activation/phosphorylation of STAT3 caused by IL-6 correct behavioral deficits in social interaction. It reduces pro-inflammatory cytokines in the CNS of C57BL/6 mice (78). Diosmin (10 mg/kg) can reduce behavioral deficits in social interaction by inhibiting the IL-6-induced JAK2/STAT3 pathway in MIA/adult offspring. Besides being safe, diosmin may have benefits for autism spectrum disorders (76).

## Effects of hesperidin and diosmin on depression

Depression is a common, recurrent, and life-debilitating chronic disorder (79). The neuropsychiatric disorder of depression is associated with decreased energy, loss of appetite, persistent negative emotions, suicidal thoughts, and sleep disorders (80). In the pathophysiology of depression, lack of neurotrophic factors, abnormal neuroplasticity, excessive inflammatory response (81), and oxidative stress are momentous (79). Enhanced levels of neuroinflammatory cytokines (such as IL-1β, TNF-α, and IL-6) are observed in individuals with depression. In patients with depression, there is an enhancement in the density of microglial cells in the frontal cortex (80).

Hesperidin (20, 50, and 100 mg/kg orally for 28 days) reduces depression-like behaviors and expression of IL-1β, IL-6, TNF-α, NLRP3, caspase-1 in the prefrontal cortex and microglia in chronic unpredictable mild stress (CUMS)-induced rats (80). Also, hesperidin (100 and 200 mg/kg orally three times a week for three weeks) reduces the levels of inflammatory factors in CUMS-induced mice through the HMGB1/RAGE/NF-kB signaling pathway and BDNF/TrkB pathway and shows antidepressant effects (81). Research suggests that pyroptosis (a form of cell death) is associated with pathophysiological signaling pathways in major depressive disorder. Hesperidin (50 mg/kg) in CUMS depressed mice may be effective in the treatment of major depressive disorder by regulating NLRP3-induced pyroptosis (82). Chronic treatment with hesperidin (0.3 and 1 mg/kg intraperitoneally) in mice increases the levels of BDNF in the hippocampus. It inhibits the l-arginine-NO-cGMP pathway, which may have an antidepressant effect (83). Hesperidin (20, 50, and 100 mg/kg, intraperitoneally for 14 days) showed antidepressant effects in rats exposed to a single prolonged stress and may be advantageous in post-traumatic stress disorder patients (84).

Diosmin (50,100 mg/kg for seven days) suppresses the NF-κB signaling pathway in the prefrontal cortex of LPS-treated mice (the classical mouse depression model). It can also prevent the translocation of p65 to the nucleus in BV2 microglial cells treated with LPS. Thus, Diosmin’s potent antidepressant effects may be mediated by blocking the NF-κB pathway (85).

## Effects of hesperidin on anxiety

Anxiety is a mental state that is defined as a feeling of fear disproportionate to the nature of the threat and can appear along with depression. There is a connection between the etiology of anxiety and depression with conditions of oxidative stress, so using anti-oxidants to prevent and treat anxiety and depression may be advantageous. Also, in support of this point of view, the anti-oxidant effects of anti-anxiety treatments such as citalopram and other antidepressants have been studied (86).

Hesperidin at doses of 50 and 150 mg/kg taken orally for ten weeks has been shown to be beneficial in reducing anxiety-like behaviors in diabetic rats. It is believed that the anti-anxiety effects of hesperidin are due to its activation of the PKA/CREB/BDNF pathway (87). In another study, oral administration of hesperidin at a dose of 50 mg/kg for 28 days demonstrated anti-anxiety and antidepressant effects in mice exposed to neurotoxicity from 6-OHDA. These effects are thought to be achieved by reducing the production of pro-inflammatory cytokines and increasing levels of neurotrophic factors, such as neurotrophin-3, which helps protect dopaminergic nerves in the striatum (40). Therefore, hesperidin has an anxiolytic effect in rodents and can reduce acute and chronic stress. 

## Effects of hesperidin on sleep disorders and pain

Disturbances in sleep can include the inability to fall asleep, insufficient sleep, and interrupted sleep. Several factors may lead to circadian rhythm and sleep disorders (88).

Hesperidin can act as a sedative and has a synergistic effect with diazepam, but it does not appear to be a ligand for the binding site of benzodiazepines (89). Hesperidin (10 mg/kg intraperitoneally) can have a sedative effect in mice by reducing the levels of pERK 1/2 in the CNS (90). Regarding the sedative mechanism of hesperidin, the current understanding is that hesperidin is not a ligand for benzodiazepine, serotonin (5HT_1A_,5 HT_2_), glutamate (AMPA), and adenosine (A_1_) receptors. Opioid receptors may be involved in the analgesic effects of hesperidin (91). The analgesic effect of hesperidin can be blocked by naltrexone and enhanced by alprazolam. Therefore, it seems that the opioid system is involved in the sedative, analgesic, and potentiating effects of hesperidin with benzodiazepines in mice (92).

## Effects of hesperidin and diosmin on neurotoxicity caused by agricultural and industrial toxins and heavy metals

The industrial solvent carbon tetrachloride (CCl_4_) causes systemic toxicity and can cause toxicity in the brain. It is used in research to create neurotoxicity models in animals. In neurotoxicity caused by CCl_4_ in Wistar rats, hesperidin (200 mg/kg orally for eight days) can correct the increased lipid peroxidation levels and protein carbonyl content and re-regulate the altered enzymatic and non-enzymatic anti-oxidants in the brain tissue. Therefore, hesperidin can be promising in improving the neurotoxicity caused by CCl_4_ by reducing neuronal oxidative stress (93). In brain tissue toxicity caused by sodium arsenite in Sprague Dawley rats, hesperidin (100 and 200 mg/kg orally for 15 days) has anti-oxidant, anti-inflammatory, and anti-apoptotic effects. It can cause tissue protection (94). Excessive fluoride has the potential to cause toxicity in brain tissue and can cause neurological disorders and mental retardation. In neurotoxicity caused by sodium fluoride in rat brain tissue, hesperidin (100 and 200 mg/kg, orally for 14 days) lessens the levels of NF-κB, IL-1B, TNF-α, and lipid peroxidation, and the caspase-3, Bax, and Bcl-2 expression levels in rat brain tissue, it also enhances the enzyme activity of SOD, CAT, and GPx, and the level of GSH in the brain tissue, and regulates the PI3K/Akt/mTOR signaling pathway. Therefore, hesperidin can be neuroprotective in neurotoxicity caused by sodium fluoride by reducing inflammation, endoplasmic reticulum stress, autophagy, and apoptosis (95). In cadmium-induced neurotoxicity in rats, hesperidin (40 mg/kg orally for 21 days) reduces oxidative stress and neurotoxicity biomarkers such as acetylcholinesterase and monoamine oxidase levels. It has anti-oxidant and neuroprotective effects in brain tissue (96). Long-term exposure to aluminum leads to neurotoxicity, including neurobehavioral and neurochemical abnormalities. With its anti-inflammatory and anti-oxidant functions, Hesperidin (50 and 100 mg/kg for 42 days) can have neuroprotective effects in neurotoxicity, cognitive disorders, and neurochemical changes in the hippocampus caused by AlCl_3_ in mice (97). Emamectin Benzoate (EMB) is an insecticide in agriculture and veterinary medicine. EMB inhibits the nervous system and causes irreversible paralysis. In rats, exposure to EMB results in behavioral, motor, and cognitive alterations in the brain. It also diminishes anti-oxidant activity and BDNF levels while augmenting inflammatory cytokines (such as TNF-α and IL-1β). Hesperidin (100 mg/kg for eight weeks) exhibits anti-inflammatory and anti-oxidant effects and can mitigate the toxic effects of EMB (98).

Millions of people around the world are exposed to water contamination with arsenic, and this exposure can be related to several neurological disorders. In the neurotoxicity caused by sodium arsenite in rats, diosmin administered orally (50 and 100 mg/kg for 21 days) exhibits a protective effect and acts as an anti-oxidant. The beneficial effects of diosmin may be through suppression of NADPH Oxidase and its subunits (99). In the thermal processing of many food products, such as meat products, fried potatoes, bread, cereals, cookies, and coffee, acrylamide may be formed and cause oxidative stress and toxicity in several organs, including the brain. With their anti-oxidant effects, hesperidin (10 mg/kg for 14 days) and diosmin (10 mg/kg for 14 days) can shield rats against oxidative stress, lipid peroxidation, and DNA damage caused by acrylamide toxicity (100).

## Conclusion

Diosmin and hesperidin are flavonoids derived from citrus fruits. These two flavonoids are available as a combination in the pharmaceutical market under purified micronized flavonoid fraction (Daflon). Diosmin and hesperidin are used as food supplements, phytopharmaceuticals, and nutraceuticals, and they are abundant in citrus fruits. Diosmin flavone glycoside is usually known for its neuroprotective properties. Hesperidin exhibits anti-oxidant, anti-inflammatory, and anti-cancer functions and protects against DNA damage and lipid peroxidation. Diosmin and hesperidin, either alone or in combination, have beneficial effects in reducing neurodegenerative symptoms. The flavonoids hesperidin and diosmin, due to their anti-oxidant, anti-inflammatory, and anti-cancer properties, neuroprotection through different signaling pathways, and very low toxicity, can be suitable choices for treatment in various nervous system diseases and neurotoxicity. Our goal in this review was to investigate the beneficial effects of these two widely used flavonoids (hesperidin and diosmin) on various toxicities and disorders of the nervous system. Future research can also explore the positive impact of flavonoids on various organs and affected signaling pathways.

## Declaration

We have not used any AI tools to prepare this manuscript.
